# Lipid Profile and Management of Dyslipidemias in Pregnancy

**DOI:** 10.3390/jcdd12110445

**Published:** 2025-11-16

**Authors:** Constantine E. Kosmas, Loukianos S. Rallidis, Ioannis Hoursalas, Eleni-Angeliki Zoumi, Christina E. Kostara

**Affiliations:** 1 2nd Department of Cardiology, National & Kapodistrian University of Athens, 12462 Athens, Greece; cekosmas1@gmail.com (C.E.K.); lrallidis@gmail.com (L.S.R.); 2 School of Medicine, European University Cyprus, Nicosia 2404, Cyprus; ihoursal@otenet.gr; 3 Guy’s & St. Thomas’ NHS Foundation Trust, London SE1 9RT, UK; eazoumi@yahoo.gr; 4 Laboratory of Clinical Chemistry, Faculty of Medicine, School of Health Sciences, University of Ioannina, 45110 Ioannina, Greece

**Keywords:** pregnancy, dyslipidemia, lipid profile, lifestyle interventions, bile acid sequestrants, fibric acid derivatives, omega-3 fatty acids, statins, PCSK9, lipoprotein apheresis

## Abstract

Dyslipidemia is a significant risk factor for atherosclerotic cardiovascular disease (ASCVD). Abnormal maternal lipid profiles in pregnancy are associated with pregnancy complications including preeclampsia, gestational diabetes, and pre-term delivery as well as increased ASCVD risk for both mother and child. Dyslipidemia management remains a central tenet for atherosclerotic cardiovascular disease prevention for women who are thinking about becoming pregnant or are already pregnant. Effective individualized guidance and multidisciplinary lifestyle/dietary strategies, along with targeted pharmacological interventions, are required for the successful management of lipid disorders in pregnancy in order to optimize outcomes. This review discusses the physiological changes occurring in lipid metabolism during pregnancy and provides an overview of the current strategies for managing dyslipidemia during pregnancy, with a special focus on consideration of pharmacological therapy.

## 1. Introduction

Dyslipidemia is characterized by abnormal levels of lipids in the bloodstream and represents the major risk factor for atherosclerotic cardiovascular disease (ASCVD), being highly predominant in the US population [1]. The majority of dyslipidemias are associated with modifiable dietary and lifestyle factors, comorbid preexisting conditions, including diabetes and hypertension, and to a lesser extent with genetic predispositions. Transient alterations in lipid levels occur during different physiological stages throughout life. These alterations in lipid profile are observed in childhood, adolescence, as well as during the biological aging process (i.e., menopausal period for women), driven by hormonal changes and environmental factors [2,3,4]. In this regard, during the physiological period of pregnancy, lipid metabolism undergoes significant alterations, driven mainly by hormonal fluctuations to support fetal growth and development.

The supraphysiological changes in total cholesterol, LDL-cholesterol, and triglycerides during pregnancy can increase the risk of developing preeclampsia, gestational diabetes and susceptibility to atherosclerosis in later life, and can lead to significant complications in women with a history of (atherosclerotic cardiovascular disease) ASCVD or pre-existing derangements in lipids [5,6]. Furthermore, maternal hypercholesterolemia can promote the development of premature atherosclerosis, dyslipidemia, and ASCVD in the offspring via complex mechanisms such as genetic programming [7,8,9]. Despite the proved association of the aforementioned adverse events and dyslipidemia during pregnancy, the professional society guidelines from the European Society of Cardiology (ESC) and the European Atherosclerosis Society (EAS) in 2018 did not fully delve into the topic of management of dyslipidemia and cardiovascular disease in pregnancy [10,11].

Early and personalized multidisciplinary care during pregnancy is necessary to improve cardiovascular outcomes and to reduce maternal mortality. To achieve target lipid levels a multifaceted approach is required, including lifestyle modifications, dietary adjustments, and potentially medical therapy [12]. In this review, we describe the alterations in lipid metabolism in uncomplicated pregnancy, and we summarize the literature on the management of dyslipidemia during pregnancy including lifestyle/dietary strategies and pharmacological interventions.

## 2. Lipid Metabolism During Normal Pregnancy

**Figure 1:**
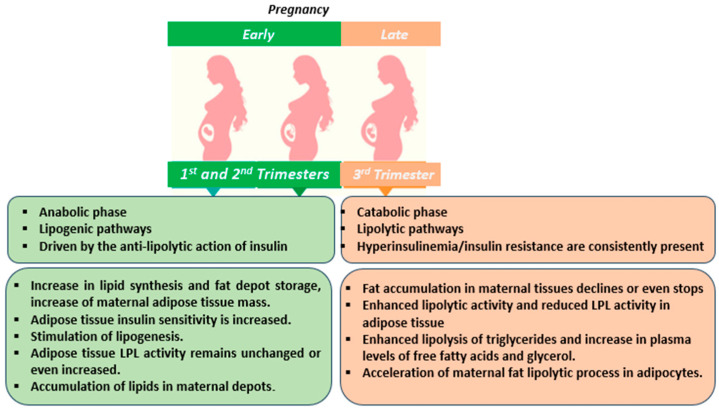
Lipid metabolism during normal pregnancy.

Throughout an uncomplicated or “normal” pregnancy, physiological changes occur in maternal metabolism in order to provide sufficient energy and nutrients for the growth and development of the fetus. With regard to lipid metabolism, the balance between lipid synthesis and breakdown is disturbed and leads into two distinct metabolic phases: an anabolic phase that presents during the first and second trimester (early pregnancy) and a catabolic one that takes place during the third trimester (late pregnancy) (Figure 1) [13]. These two phases are associated with metabolic adaptations of adipocyte functions, resulting in a predominance of lipogenic and lipolytic pathways, respectively. The net anabolic phase seems to be driven by the anti-lipolytic action of insulin, whereas in the catabolic phase both hyperinsulinemia and insulin resistance are consistently present. Additionally, maternal hyperlipidemia is normally developed during the last third of gestation, as described in detail below.

### 2.1. Maternal Adipose Tissue Metabolism

***Anabolic phase:*** During the first two trimesters of gestation, there is an increase in lipid synthesis and fat depot storage that leads to a rapid increase in maternal adipose tissue mass [14]. This enhanced lipogenesis is attributable in part to maternal hyperphagia [15], as well as to dynamic metabolic and endocrine changes that normally occur during this stage of pregnancy.

Adipose tissue insulin sensitivity is increased thus stimulating lipogenesis. The activity of adipose tissue lipoprotein lipase (LPL) remains unchanged or even increased. The LPL enzyme is present in the capillary endothelium of extrahepatic tissues, being especially active in adipose tissue, and catalyzes the hydrolysis of circulating triacylglycerols that are carried in triglyceride-rich lipoproteins such as chylomicrons and very low-density lipoproteins (VLDL). The resulting products, non-esterified (free) fatty acids and glycerol, are mostly taken up by the adipose tissue for re-synthesis of triglycerides, which are accumulated in maternal fat depots. Additionally, pregnancy normally induces endocrine changes, including increased levels of maternal estrogen, progesterone, cortisol, leptin, and prolactin, which result in inhibition of lipolysis and promotion of both lipogenesis and fat accumulation [16].

Overall, the aforementioned metabolic environment favors the accumulation of lipids in maternal depots during early pregnancy in order to meet the lipid requirements of the developing fetus in late pregnancy.

***Catabolic phase:*** The anabolic phase observed in maternal adipose tissue during the first two trimesters of gestation gradually shifts to a net catabolic state that characterizes late pregnancy. In this state of metabolism, fat accumulation in maternal tissues declines or even stops due to enhanced lipolytic activity and reduced LPL activity in adipose tissue [17]. These dynamic metabolic changes are favored by the maternal insulin-resistant state that characterizes the last trimester of pregnancy. Insulin inhibits adipose tissue lipolysis and hepatic gluconeogenesis and ketogenesis and enhances adipose tissue LPL activity. Additionally, pregnancy-induced increase in hormones’ levels, including progesterone, cortisol, and prolactin, enhance insulin resistance in peripheral tissues, such as the adipose tissue, during the second trimester onward, with the highest insulin resistance noted at the third trimester of gestation. Placental growth hormone (GH-V) also induces maternal insulin resistance, whereas adipokines, such as leptin and adiponectin, also mediate insulin resistance.

The enhanced lipolysis of triglycerides, which are transferred by chylomicrons and liver-derived VLDLs, as well as those that are stored in adipocytes, results in an increase in plasma levels of free fatty acids and glycerol. Free fatty acids are transported to the growing fetus via placenta, whereas glycerol is mainly transported to the maternal liver. There, after being converted into their active forms (acyl-CoA and glycerol-3-phosphate), they are re-esterified for the synthesis of triacylglycerols. The latter are transported through the bloodstream as the main hydrophobic lipid component of VLDL particles’ core. In addition, the increased maternal plasma glycerol levels enhance gluconeogenesis, since glycerol is preferentially used as a substrate for glucose, which is easily transferred via the placenta to the fetus. The reduced adipose tissue LPL activity [18] leads to reduction in the hydrolysis and tissue uptake of triglycerides from triglyceride-rich lipoproteins, thus contributing to the development of maternal hyperlipidemia.

The acceleration of maternal fat lipolytic process in adipocytes and the subsequent increment in plasma levels of free fatty acids and glycerol is also stimulated by the third-trimester increase in placental hormones with lipolytic effects and release of catecholamines in response to maternal hypoglycemia [19].

### 2.2. Maternal Hyperlipidemia

The net catabolic condition in maternal adipose tissue is associated with maternal hyperlipidemia entailing elevated plasma levels of lipids, mainly triglycerides, and to a lesser extent cholesterol and phospholipids. The ratio of triglycerides to cholesterol remains stable/unchanged in VLDLs but increases in both LDLs and HDLs.

It is well known that triglycerides are the predominant lipids in VLDLs, although they are also contained in both LDLs and HDLs but in lower proportion, especially in HDLs. In the third trimester, the abundance of plasma VLDL particles is high as a consequence of their enhanced production by the liver [20] and decreased removal from the circulation due to low LPL activity in adipose tissue [18]. The hepatic production of VLDLs is enhanced due to the increased lipolysis of triglycerides in adipocytes. The resulting free fatty acids are transported to the liver to form VLDLs. Apart from the increase in VLDL-triglycerides, both LDLs and HDLs are enriched in triglycerides due to an increased activity of cholesteryl ester transfer protein (CETP) [21]. This enzyme promotes the transfer of cholesteryl esters from atheroprotective HDLs to atherogenic apolipoprotein B–containing lipoproteins, including VLDLs, VLDL remnants, IDLs, and LDLs. This, together with a decrease in hepatic lipase activity that presents during late pregnancy [22], explains the enrichment of LDLs and HDLs in triglycerides.

The increase in plasma cholesterol levels is likely due to increased rates of hepatic cholesterol synthesis. Additionally, changes in pregnancy-related hormones may be responsible for the metabolic interactions that result in the development of maternal hypertriglyceridemia during late pregnancy. The high plasma estrogen concentrations in the third trimester probably contribute to these lipid derangements because estrogens are known to stimulate hepatic lipogenesis and VLDL production and to decrease hepatic lipase activity. Overall, the aforementioned pathophysiological mechanisms are synergistically responsible for the altered lipid profile in late pregnancy.

## 3. Lipid Profile During Normal Pregnancy

Lipids sustain the development and growth of the fetus and are crucial structural and bioactive components for the maintenance of placental function. They are provided to the fetus via exogenous pathways (exogenous lipids) or are produced de novo by the fetus (endogenous lipids). For example, a significant portion of cholesterol that is essential for fetal development is produced de novo by the fetus. It is an indispensable lipid component of cell membranes and serves as a building block for the synthesis of various steroid hormones, vitamin D, and bile acids. Triglycerides together with glucose are the major sources of fetal energy.

Early in pregnancy, lipids are transported to the fetus via the yolk sac, whereas, when approaching 8 weeks of gestation, their transport is mediated via placenta with a highly regulated and dynamic process as gestational age increases. Apart from its role as lipid transporter to the fetal compartment, placenta converts maternal lipids to free fatty acids for uptake and processing by trophoblast cells for metabolic demands, to produce hormones for the maintenance of pregnancy, and to transfer them to the developing fetus. In early pregnancy, robust lipid uptake is vital to meet the high energy demands required for the growth of placenta and the development of embryonic organ systems, whereas in the later stages of pregnancy lipids are required for the development of nervous system.

Following conception, there is a decrease in the levels of TG, TC and LDL-C to a nadir at the second gestational month. The levels then increase with a peak at the delivery month. As an uncomplicated pregnancy progresses, serum lipid parameters are normally increased as gestation proceeds, and become more pronounced during the third trimester in order to be geared to benefit fetal development and growth (Table 1). The magnitude of these changes during pregnancy are affected by many factors, including pre-pregnancy lipid levels and body mass index (BMI), age, diet, and ethnicity [23,24,25].

Total and LDL-cholesterol levels can increase approximately 30–50%, while triglycerides may increase up to 200–400% [26]. Compared to other lipid parameters, triglycerides increase disproportionately during pregnancy as their levels can more-than-double during the third trimester. The increase in triglycerides levels is mainly due to increased hepatic synthesis, which triples from the 14th week of pregnancy to the last weeks. Therefore, the increase in triglycerides as gestational age increases leads to an increase in VLDLs through which they are transported. Increased lipolysis of adipose tissue leads to an increase in fatty acids and glycerol. The latter are transported to the liver and are re-esterified to form triglycerides, which are ultimately transported via VLDLs. The insulin-resistant state of pregnancy contributes to the increased production of VLDLs.

HDL-cholesterol levels and apolipoprotein A1 (apoA1) increase 20–40% from early pregnancy onwards and plateau around 20–24 weeks [27]. Especially, HDL-cholesterol increases gradually from the 14th week of pregnancy until the 28th week due to an increase in the HDL2 subfraction. The latter can reach 40% due to the increase in estrogen levels, which favors the hepatic production of ApoA1, and the reduction in the activity of hepatic lipase, which is responsible for the hydrolysis of HDL2 into smaller HDL3, which are removed from the circulation more quickly.

Lipoprotein (a) [Lp(a)] levels increase steadily between the 10th and 35th weeks in normal pregnancy (median increase 170%, range 117–340%), show a small decrease before delivery and return to pre-first trimester levels in the 3–5 months postpartum.

It should be mentioned that in uncomplicated or “normal” pregnancies, neither total cholesterol nor triglycerides concentrations exceed 250 mg/dL at any time during pregnancy [28,29]. Over the three months post-partum, the level of major lipoproteins and lipids declines toward pre-pregnancy levels [30].

## 4. Management of Lipid Disorders During Pregnancy

### 4.1. Lipid Profile Screening

According to the 2018 American Heart Association (AHA)/American College of Cardiology (ACC)/Multisociety Guideline on the Management of Blood Cholesterol, lipid profile screening should be started at the age of 20 years for all adults including women of reproductive age [31]. The 2021 ESC guidelines [32] do not provide a single mandatory age for routine lipid testing but recommend a stepwise individualized approach based on risk factors. Despite national screening guidelines to perform a lipid profile test in children and young adults, many reproductive-age women have not undergone lipid screening. Additionally, up to now there are no specific lipid screening guidelines regarding when to screen women considering pregnancy and how often to screen them during pregnancy, thus, it should be individualized.

Preconception routine lipid panel screening could help identify women at risk for ASCVD and provide opportunities for optimal maternal and fetal health outcomes with appropriate interventions. Golwala et al. [33] incorporated a non-fasting lipid panel into routine prenatal care among obstetricians at a single academic clinic. They assessed 445 pregnant women, among whom 236 (66%) patients completed lipid testing, showing abnormal levels in 59 (25%) patients. Implementation of lipid screening as part of routine prenatal care during the first trimester may play a crucial role in timely diagnosis and management of lipid disorders in women of reproductive age [33].

Of note, women with advanced maternal age, a personal or family history of lipid disturbances, premature ASCVD, and/or with coexisting comorbidities, including chronic hypertension, obesity, diabetes, and/or polycystic ovary syndrome, who are planning to become pregnant are recommended to be prioritized for lipid screening before pregnancy.

### 4.2. Dietary and Lifestyle Interventions

Nutrition is considered one of the most important factors that can affect embryonic and fetal development, as well as the health of both mother and her offspring [34]. The maternal nutritional status before conception and/or during the first weeks of pregnancy can influence critical processes of fetal growth that begins early in pregnancy. Fetal growth is largely determined by nutrient supply, which is dependent upon the maternal nutritional status and dietary intake, as well as placental nutrient transport. The placenta acts as a selective barrier, controlling the transfer of nutrients and influencing fetal growth and development. Especially in the later stages of pregnancy, the placenta plays a crucial role in regulating nutrient supply to the developing fetus, adapting to both maternal nutrient availability and fetal demands. In addition, maternal endocrine and metabolic adaptations that normally occur early in pregnancy play a crucial role in supplying the rapidly developing fetus with essential nutrients in the later stages of pregnancy [35,36].

Dietary and lifestyle intervention is the cornerstone for the treatment of lipid disorders in the general population and the foundation for the prevention and management of atherosclerotic cardiovascular disease (ASCVD). There are no specific nutritional guidelines for managing dyslipidemia for women in pregnancy and lactation. The evidence-based nutrition interventions recommended for non-pregnant women with dyslipidemia are applied to pregnant women with dyslipidemia, acknowledging the constraints imposed by pregnancy. However, it has been shown that only 0.1% of pregnant women follow an optimal dietary pattern, thus counseling on personalized nutrition approaches is recommended. Yamamoto A et al. showed that pregnant women were significantly less likely to receive diet and exercise counseling than non-pregnant women (17.9% vs. 22.6%) [37].

In general, pregnant women with lipid disorders should avoid excessively restrictive diets and should be encouraged to consume a cardioprotective dietary pattern to ensure a healthy fetal growth as well as protection of both fetus and mother from cardiometabolic diseases throughout the duration of their life. The European Food Information Council (Eufic) provide guidelines for a healthy pregnancy diet, recommending a variety of fruits, vegetables, whole grains, lean protein (lean meat, seafood, legumes, nuts, seeds) and dairy, while limiting raw and undercooked meats and seafood, high mercury fish, unpasteurized dairy, and alcohol. Key recommendations include safe food handling, increasing fluid intake ensuring sufficient folate and vitamin D and limited caffeine [38].

From a nutritional point of view, among different recommended dietary patterns, the Mediterranean-style dietary pattern represents the best dietary model in pregnancy [39]. Although this pattern may vary slightly from country to country, it provides important health benefits for the metabolic status of both mother and fetus. Studies have shown that greater maternal adherence to the Mediterranean diet was associated with improved maternal lipid profile during pregnancy and postpartum [40,41], lower risk of postpartum depression, lower incidence of gestational diabetes mellitus and child adiposity [42,43], reduced risk of preeclampsia [44], and better neurological development in the newborn [45]. DASH diet may also be beneficial in pregnancy [46].

Regular exercise plays a crucial role in mitigating cardiometabolic risk and in ASCVD prevention. For pregnancy, studies regarding exercise, conducted in the 1970s and 1980s, focused primarily on potential adverse health outcomes for pregnant women, due to limited available data on how pregnant women respond to exercise and on its effects on pregnancy. Since then, published data support that regular physical activity during pregnancy can confer numerous benefits, including decreased incidence of gestational diabetes, hypertension, and excess weight gain and weight retention in the postpartum period, as well as decreased symptoms of postpartum depression [47,48,49,50]. Thus, after careful clinical evaluation, women with uncomplicated pregnancies should be encouraged to participate in physical activity with preferred type of exercise including aerobic and strength-training activities before, during, and after pregnancy.

Overall, dietary and lifestyle intervention is an indispensable component of the multidisciplinary approach to treating pregnant women with lipid metabolism disorders and preventing complications associated with elevated levels of triglycerides and cholesterol.

### 4.3. Pharmacological Treatments in Pregnancy

There are many pharmacological approaches to intervene in the management of dyslipidemia; however, in pregnancy only a few are recommended as discussed below and in Table 2. 

**Bile acid sequestrants:** Bile acid sequestrants (BAS) including cholestyramine, colestipol, and colesevelam are approved by the Food and Drug Administration (FDA) in combination with restriction of dietary saturated and trans-fatty acids to manage hypercholesterolemia. Indeed, BAS bind bile acids in the intestinal lumen and form an insoluble complex thus preventing their reabsorption into the maternal systemic circulation. The enterohepatic circulation of bile acids and their re-entry into hepatocytes via the portal circulation are interrupted. Thus, as the levels of bile acids in hepatocytes decrease, the metabolic pathway for bile acid production in the liver is activated. As a result, more hepatic cholesterol converts to bile acids due to the disinhibition of cholesterol 7-alpha hydroxylase, the rate-limiting step in bile acid production. This in turn increases hepatic cholesterol biosynthesis rate and upregulates LDL receptors on the surface of hepatocytes, which results in increased catabolism of atherogenic LDL particles and therefore in a decrease in total and LDL cholesterol levels.

BAS causes a significant dose-dependent reduction in total and LDL-cholesterol levels. When used as monotherapy, they cause a reduction in LDL-cholesterol levels by approximately 20–30%, while when combined with statins, they cause an additional reduction of approximately 10% [51]. However, the increased hepatic synthesis of cholesterol leads to increased secretion of VLDL lipoprotein particles and consequently to elevated plasma triglyceride levels. For this reason, these lipid-lowering drugs are contraindicated as monotherapy in patients with triglyceride levels higher than 200 mg/dL and in those with dysbetalipoproteinemia.

To date, BAS is pregnancy category C and considered the safest lipid-lowering agents for use in pregnancy. However, limited data is available on its use in pregnant women. The most common adverse effects of BAS include gastrointestinal side effects such as constipation, abdominal pain, loss of appetite, indigestion, bloating, vomiting, and heartburn, which may exacerbate symptoms already present during normal pregnancy. Moreover, BAS can induce malabsorption of fat-soluble vitamins such as vitamins A, D, E and K, as well as folic acid, and other medications that may be important for fetal and maternal health. However, the lack of systemic absorption limits the potential for BAS to impair vitamin absorption in the infant. Among BAS, data support that colesevelam seems to be the better-tolerated resin.

**Fibric acid derivatives**: Although CVD risk is increased when fasting triglycerides are >150 mg/dL [52], the use of drugs to lower triglyceride levels including the fibric acid derivatives, gemfibrozil and fenofibrate, may only be considered in high-risk patients when triglycerides are >200 mg/dL. The mechanism by which fibric acid derivatives interfere with lipid metabolism includes the activation of transcriptional factors known as peroxisome proliferator-activated receptors (PPAR), especially the PPAR-a form that is expressed in liver [53].

Findings from animal studies have shown potential fibrate-induced complications (delayed delivery, reduced birth weight, increased post-implantation loss, skeletal and visceral abnormalities, abortions, and fetal deaths), thus classifying both fenofibrate and gemfibrozil as pregnancy category C drugs. Soria et al. proposed the opposite metabolic response to fenofibrate treatment in pregnant and virgin rats are a consequence of both enhanced liver capability for VLDL triglyceride production and a rebound response to the drug in the latter [54].

Concerning the safety of fibric acid derivatives in pregnancy, only limited data from human studies exist, derived mainly from case reports. It has been reported that fibric acid derivatives may be associated with the development of gallstones during pregnancy; however, case reports have reported no adverse effects on the fetus. Sunman et al. published a case report where the embryo had been exposed to fenofibrate from the beginning of fertilization [55]. According to the authors, the 30-year-old female delivered a healthy male infant at 36 weeks of gestation. Fetal ultrasound performed routinely during each trimester showed normal fetal growth with no malformation. The authors concluded that in this case no harmful effects on fetal development were observed after exposure to fenofibrate during organogenesis [55]. Whitten et al. reported on a case of hypertriglyceridemia-induced pancreatitis in a woman presenting at 32 weeks of gestational age [56]. Since hypertriglyceridemia was not managed with nutrition and lifestyle interventions, medical treatment was necessary. According to the authors fenofibrate was used safely and successfully during pregnancy, the recurrence of pancreatitis during the pregnancy was avoided, and a healthy neonate was delivered at 35 weeks of gestation [56].

Since fenofibrate was found to be present in rat milk, it is presumed to be likely present in human milk; therefore, lactating women should not breastfeed during treatment with fenofibrate. In a lactating woman receiving fenofibrate, it is recommended to wait for at least five days after the final dose before resuming breastfeeding. The AHA Scientific Statement for Cardiovascular Considerations in Caring for Pregnant Patients proposes the consideration of fenofibrate or gemfibrozil in the second trimester in pregnant women with very high triglycerides levels (>500 mg/dL) [30]. Also, the AHA/American College of Obstetricians and Gynecologists (ACOG) Presidential Advisory states that pregnant patients with a history of pancreatitis may benefit from the use of fenofibrate when triglyceride levels are >1000 mg/dL [57]. The 2021 ESC guidelines do not provide extensive details on lipid management in pregnancy [32].

**Omega-3 fatty acids:** From the structural point of view, omega-3 fatty acids are polyunsaturated fatty acids (PUFAs) with at least one double bond between the third and fourth omega-end carbon. Omega-3 PUFAs that are derived from the oil of fish and other seafood, have been approved by FDA as a safe and effective strategy to manage severely elevated TG levels (≥500 mg/dL) and to reduce cardiovascular events. They cause a dose-dependent reduction in triglyceride levels by 20–30% and a slight decrease in non-HDL-C and apoB levels.

While limited data exist on the safety of prescription omega-3 fatty acids for lipid disorders in pregnant women, some evidence suggests potential benefits including reduced risk of preterm birth, early preterm birth and perinatal death. Nguyen et al. reported on management of two cases with severe gestational hypertriglyceridemia [58]. The authors concluded that omega-3 fatty acids may be effective and safe to treat pregnant women with severe hypertriglyceridemia since triglyceride levels were reduced to less than 500 mg/dL until delivery of healthy babies in both cases [58].

Middleton P et al. conducted a meta-analysis including 70 randomized controlled trials (RCTs) in order to assess the effects of omega-3 PUFA, as supplements or as dietary additions, during pregnancy on maternal, perinatal, and neonatal outcomes and longer-term outcomes for mother and child [59]. Beyond their triglyceride-lowering effects, the use of omega-3 PUFAs during pregnancy reduced the risk of preterm birth (<37 weeks), early preterm birth (<34 weeks), perinatal death, and low birthweight (LBW) babies. Moreover, according to the authors, little or no difference in small-for-gestational age or intrauterine growth restriction was observed. However, it is important to note that omega-3 PUFAs may slightly increase the risk of large-for-gestational- age (LGA) babies [59].

Omega-3 fatty acids in doses up to 4 g per day constitute an affective therapeutic option for patients with severe hypertriglyceridemia, particularly those at high risk of pancreatitis (individuals with TG levels > 500 mg/dL who are asymptomatic or have a history of pancreatitis), even when administrated in combination with fenofibrate, provided that the clinical judgment is that the benefits outweigh the risks [12,60,61].

**Statins:** Statins are the standard-of-care for the treatment of dyslipidemia exhibiting well-established benefits in primary and secondary prevention of cardiovascular events. They inhibit the rate-limiting enzyme in cholesterol synthesis—hydroxymethylglutaryl-coenzyme A (HMG-CoA) reductase, resulting in reduced intracellular cholesterol synthesis and circulating LDL-cholesterol levels, a well-established risk factor for cardiovascular disease. HMG-CoA reductase is the enzyme responsible for the conversion of HMG-CoA to mevalonate in the mevalonic acid pathway of cholesterol biosynthesis.

Despite the well-known lipid-lowering effects of statins, there has been historic concern surrounding their use in pregnancy. Statin treatment during pregnancy and lactation was not recommended (FDA old category X). This recommendation mainly relies on data derived from animal studies, which demonstrated a treatment-related increase in the incidence of gastroschisis and malformations of skeleton in rats [62] and a decrease in the fetal body weight in rabbits and rats [63]. It is worth noting that the dose of statin that was used in animal studies exceeded typical human prescription usage [62,63].

Human case studies have also suggested potential adverse birth outcomes following statin exposure. An early analysis was conducted by Manson et al. who reviewed 134 cases of first trimester exposure to lovastatin or simvastatin with known pregnancy outcomes [64]. Among those 134 pregnant women exposed to statin therapy, the proportion of normal outcomes was 85%, congenital anomalies 4.0%, spontaneous abortions 8.0%, fetal deaths/stillbirths 1.0%, and miscellaneous adverse outcomes 2.0%. The authors concluded that there was no clear relationship between exposure to statin therapy and adverse pregnancy outcomes.

Edison et al. published a study in the New England Journal of Medicine suggesting possible statin-related effects on embryogenesis [65]. A total of 178 cases of first-trimester statin exposure reported to the Food and Drug Administration (FDA) for patterns suggesting possible drug-related effects on embryogenesis. After the exclusion of cases including first trimester elective or spontaneous abortions, pregnancy loss due to maternal illness, fetal genetic disorders, transient neonatal disorders, or loss to follow-up, the remaining 52 cases were included in the study. Among them, the authors reported 20 cases of malformation in infants (limb deficiencies and central nervous system defects). Of note, the aforementioned adverse outcomes at birth were reported in mothers who were exposed to lipophilic statin in the first trimester, whereas no malformations were reported among 14 infants exposed to hydrophilic statin (pravastatin) potentially due to low tissue penetration [65].

Pollack et al. determined the frequency of adverse outcomes after maternal exposure to simvastatin and/or lovastatin during pregnancy [66]. The authors identified 477 reports (386 prospective and 91 retrospective) with 225 prospective outcomes reported (154 live born infants, 49 elective abortions, 18 spontaneous abortions, and 4 fetal deaths). Among the prospective cases, six congenital anomalies (chromosomal translocation, trisomy 18, hypospadias, duodenal atresia, cleft lip, and skin tag) were reported. It is worth noting that the rate of congenital anomalies (congenital anomalies/live births plus fetal deaths) was 3.8%, which is similar to the background population rate (3.2%; relative ratio, 1.21; 95% 1-sided upper confidence interval [2], 2.02). Among the retrospective cases, 13 retrospective reports describe a range of congenital anomalies. The authors concluded that there was no evidence to support an increased risk of congenital anomalies in women who were exposed to statin therapy during their pregnancy (either the prospective or retrospective reports) [66].

Petersen et al. identified 13 cases of congenital anomalies (seven congenital heart defects, two cleft lip or palate defects, two neural tube defects, and two other miscellaneous defects) from the National Birth Defects Prevention Study (NBDPS) and nine birth defects (five congenital heart defects, two cleft lift lip defects, and two other miscellaneous defects) from the Sloan Epidemiology Center Birth Defects Study (SEC-BDS) [67]. Of note, 11 of the total 22 cases occurred in mothers with pre-existing diabetes. The researchers concluded that they could not determine either the risks or safety of statin therapy in pregnancy [67].

These case studies that reported congenital anomalies associated with exposure to statins were followed by a number of cohort studies of statin exposure in pregnancy that failed to show an increase in teratogenic risk.

In a prospective, observational cohort study, which included 64 pregnant women exposed to statins (atorvastatin, simvastatin, pravastatin, rosuvastatin) during the first trimester and 64 women group without exposure to known teratogens, no difference was observed in the rate of major malformations between the two groups 2.2% vs. 1.9% (1/46 live birth in statin group vs. 1/52 live birth in control group, *p* = 0.93). The researchers did not find statistical differences between the statin and control group concerning live births (71.9% vs. 81.2%), spontaneous abortions (14: 21.9% vs. 11: 17.2%), therapeutic abortions (3: 4.7% vs. 0: 0%), and stillbirths (1: 1.5% vs. 1: 1.6%) [68]. Winterfeld et al. performed a multicenter observational prospective controlled study that did not detect a teratogenic effect of statins [69]. In this study, pregnant women exposed to statins during first trimester were followed up prospectively. Data analysis did not reveal a statistically significant difference in pregnancy outcomes (rate of major birth defects, median gestational age at birth and birth weight) between statin-exposed and matched non-exposed pregnancies. It is worth noting though that premature birth was more frequent in exposed to statins pregnancies (16.1% versus 8.5%; OR 2.1, 95% CI 1.1–3.8, *p* = 0.019) [69].

In another cohort study involving 886,996 completed pregnancies, the authors examined the risk of the major congenital malformations and organ specific malformations in offspring associated with maternal statin use [70]. Of these pregnancies, 1152 (0.13%) women used a statin during the first trimester. In the initial unadjusted analysis, the prevalence of malformations in the offsprings was 6.34% compared with 3.55% in those of non-statin exposed pregnant women in the first trimester (relative risk (RR) 1.79, 95% confidence interval (CI) 1.43 to 2.23). However, after adjusting for confounders, particularly pre-existing diabetes, this risk was decreased (1.07, 0.85 to 1.37). Additionally, according to the authors, there were also no statistically significant increases in any of the organ specific malformations assessed after accounting for confounders. The researchers did not find significant teratogenic effects associated with maternal use of statins in the first trimester [70].

A population-based pregnancy registry study published by Ofori et al. [71] examined the association between statin use in early pregnancy and the incidence of congenital anomalies in live births. They enrolled 288 pregnant women that were assigned into three groups: women prescribed statins in the first trimester (group A), women prescribed fibrate/nicotinic acid in the first trimester (group B) and those used statins between one year and one month before conception, but not during pregnancy (group C). The results demonstrated that among women with a live birth, the rate of congenital anomalies was 3/64 in group A (4.69%; 95% CI 1.00, 13.69), 3/14 in group B (21.43%; 95% CI 4.41, 62.57) and 7/67 in group C (10.45%; 95% CI 4.19, 21.53). The adjusted OR for congenital anomalies in group A compared with group C was 0.36 (95% CI 0.06, 2.18). Thus, the authors concluded that they did not detect a pattern in fetal congenital anomalies or evidence of an increased risk in the live-born infants of women filling prescriptions for statins in the first trimester of pregnancy [71].

Colvin et al. [72] linked a pharmaceutical claims database in Western Australia from 2002 to 2005 (106,074 birth events) with a birth defects registry to investigate birth defect rates of suspected teratogens. The authors identified records of births, whose mothers were dispensed medicines in categories D or X of the Australian ADEC pregnancy risk category. Among the records of births, the researchers identified 51 records of live births whose mothers have been exposed to atorvastatin (n = 33) and simvastatin (n = 18) during the first trimester; however, no congenital anomalies were reported in the study.

Pregnant women with familial hypercholesterolemia are likely to be exposed to an accelerated atherosclerosis risk and thus may be at a significantly increased risk of premature CVD. Toleikyte I et al. conducted a registry-based study that enrolled 1869 women with familial hypercholesterolemia and about 2 million persons from the general population of Norway [73]. The registry match resulted in the analysis of 2319 births of 1093 women with heterozygous familial hypercholesterolemia. According to the authors, nineteen pregnant women used lipid-lowering drugs (15 used statins, 3 used bile acid sequestrants, and 1 used a combination of them). Data demonstrated that pregnant women with familial hypercholesterolemia exposed to lipid-lowering drugs do not appear to have a higher risk of preterm delivery or of having infants with low birth weight or congenital malformations than women in general [73].

A few years later, Botha et al. investigated the effect of gestational statin exposure in genotypically confirmed female patient with homozygous familial hypercholesterolemia [74]. Data did not demonstrate an increased risk of teratogenic effects due to statin exposure. The authors concluded that the complications associated with pregnancy in women with homozygous familial hypercholesterolemia who used statins did not differ from those reported during pregnancies of otherwise healthy woman.

In 2014, a meta-analysis was conducted to investigate pregnancy outcome following first trimester exposure to statins [75]. In this meta-analysis, retrospective or prospective controlled studies of pregnant women exposed to a statin and studies that included a control group of women unexposed to statins were included. Data concluded that statin exposure was not associated with a significant increase in birth defects compared with control subjects (RR 1.15). However, there was a modest but significant increase in miscarriage risk (RR 1.35), and women using statins were more than twice as likely as control subjects to undergo elective terminations of pregnancy [75].

A systematic review of 16 clinical studies (5 case series, 3 cohort studies, 3 registry-based studies, 1 randomized controlled trial, and 4 systematic reviews) did not show any clear relationship of congenital anomalies with statin use in pregnancy, supporting the findings that statins are probably not teratogenic [76]. In most studies, women were exposed to statins during the first trimester; however, the duration of exposure was not clearly defined [76].

Recently, Karadas et al. conducted a meta-analysis of five cohort studies and one case–control study in order to investigate whether maternal exposure to statins is associated with increased rates of major congenital malformations and other adverse pregnancy outcomes [77]. Data analysis did not show significant increase in rate of major congenital malformations when statin-exposed group was compared with the control ([OR 1.27; 95% CI 0.80–2.04], [aOR 1.05; 95% CI 0.84–1.31]). The authors though found a significantly lower live birth rate (OR 0.60, 95% CI 0.49–0.75) and a higher spontaneous abortion rate (OR 1.36; 95% Cl 1.06–1.75) in the statin-exposed group. In addition, a significant increase in heart defect risk was observed in the statin-exposed group when unadjusted ORs were combined (OR 2.47; 95% CI 1.36–4.49). However, when adjusted ORs were used, no significant increase in heart defect risk in the statin-exposed group compared with the controls (aOR 1.24; 95% CI 0.93–1.66). The authors concluded that gestational statin exposure was not associated with a significant increase in risk of major congenital malformations, heart defects and other adverse pregnancy outcomes, except for increased spontaneous abortions and lower live birth rate, which, however, may be associated with maternal comorbidities and other unadjusted risk factors [77].

Preeclampsia is one of the most common hypertensive complications of pregnancy that affects approximately 3% to 8% of pregnancies with considerable neonatal and maternal morbidities and mortalities. Costantine et al. conducted a pilot, multicenter, double-blind, placebo-controlled, randomized trial in order to evaluate the safety of pravastatin in pregnant women at high risk of preeclampsia [78]. They enrolled women between twelve- and sixteen-weeks’ gestation, who were assigned to daily pravastatin 10 mg or placebo orally until delivery. The results of this phase I clinical trial provided preliminary safety and pharmacokinetic data regarding the use of pravastatin for preventing preeclampsia in high-risk pregnant women. The authors found no differences between the two groups in the rates of study drug side effects, congenital anomalies, other adverse or serious adverse events, and of maternal, fetal, or neonatal death. Five years later, Costantine et al. reported a follow-up, multicenter, blinded, placebo-controlled, randomized trial of 20 mg pravastatin versus placebo among pregnant women with previous preeclampsia who required delivery before 34 + 6 weeks’ gestation with the objective of evaluating the safety and pharmacokinetic parameters of pravastatin [79]. There were no significant differences between the two groups in the rates of adverse or serious adverse events, congenital anomalies, or maternal and umbilical cord blood chemistries, confirming the overall safety and favorable pregnancy outcomes for pravastatin in women at high risk for preeclampsia.

Studies using tissue cultures and animal models were conducted to investigate whether statins could reverse pathophysiological pathways underlying the development of preeclampsia and to ameliorate its phenotype [80,81,82,83].

In a recent meta-analysis of five studies including 1570 pregnant women who received either pravastatin or placebo, the role of pravastatin use prior to 20 weeks of gestation, to prevent preeclampsia was evaluated [84]. According to the authors pravastatin treatment reduced the incidence of preeclampsia by 61% and premature birth by 45%. Among the newborns, there was a 45% reduction in intrauterine growth retardation (IUGR) in the treated group, as well as a 77% reduction in those receiving neonatal intensive care unit (NICU) admissions. They concluded that prophylactic treatment with pravastatin appears to reduce risk of developing preeclampsia as well as potentially lowering risk of IUGR, preterm birth, and NICU admission in neonates [84]. Nevertheless, in another multicenter, double-blind, placebo-controlled trial, which included 1120 women with singleton pregnancies at high risk of term preeclampsia, who were randomly assigned to receive pravastatin at a dose of 20 mg/d or placebo from 35 to 37 weeks of gestation until delivery or 41 weeks, pravastatin administration in women at high risk of term preeclampsia did not reduce the incidence of delivery with preeclampsia [85].

The results of several trials suggest that pravastatin started in the second to third trimester of pregnancy is generally safe without an increase in congenital anomalies. Pravastatin was used in these trials because of its hydrophilic status and because it is a substrate of the P-glycoprotein efflux pump, theoretically limiting its ability for transplacental transfer. Furthermore, in animal models of preeclampsia, pravastatin use led to improvement of vascular profile, lowering of blood pressure, and restoration of angiogenic balance in the mother without adversely affecting offspring outcomes [86].

It should be noted that, in July 2021, the FDA removed the strongest label warning regarding statins in pregnancy (FDA 2021). Although most women are recommended to discontinue statins prior to pregnancy, this label change allows more flexibility for treatment options in pregnant women, particularly those at highest ASCVD risk, as part of shared decision-making.

**Proprotein convertase subtilisin/kexin type 9 (PCSK9) inhibitors:** Proprotein convertase subtilisin kexin 9 (PCSK9) is a pivotal enzyme that accelerates the degradation of LDL receptors intracellularly, resulting in increased plasma LDL-cholesterol levels. Since its discovery in 2003 [87], it has attracted a lot of attention as a revolutionary therapeutic target for hypercholesterolemia. The US Food and Drug Administration (FDA) have approved two monoclonal antibodies, alirocumab and evolocumab, to inhibit or reduce PCSK9 activity [88]. Recently, both the European Union and FDA have approved inclisiran, a small interfering mRNA, to inhibit intracellular PCSK9 synthesis in the liver [89].

Given that PCSK9 protein plays an important role in cellular differentiation and proliferation, it is reasonable to be concerned about the use of PCSK9 inhibitors during fetal development. Safety data on the use of monoclonal antibodies inhibiting PCSK9 in pregnancy are scarce.

Vuignier Y et al. published a case report of a 28-year-old woman with familial hypercholesterolemia, who required a three-vessel coronary artery bypass graft, drug-eluting stents, rosuvastatin, ezetimibe, and alirocumab at maximal dosage [90]. An unplanned pregnancy required an abrupt stop of all oral medications at six gestational weeks, except for aspirin. According to the authors, the data did not support a causal link between the early pregnancy exposure to PCSK9 inhibitors and the observed complete agenesis of the corpus callosum [90]. Suzuki et al. presented the first case of initiating evolocumab, a PCSK9 inhibitor, in a 34-year-old mother with heterozygous familial hypercholesterolemia [91]. Evolocumab was administered at 31 and 35 weeks of gestation. According to the authors, the planned delivery was performed at 38 + 4 weeks and both the mother and infant were discharged without any notable complications. However, the authors pointed out the need for further studies to assess the safety of evolocumab administration during pregnancy [91].

Ardissino et al. used drug-target Mendelian randomization to model the potential impact of fetal LDL-lowering, overall and through PCSK9 drug targets, on congenital malformations in approximately 1.3 million individuals [92]. The authors found that genetically proxied LDL-lowering through PCSK9 was associated with higher odds of malformations affecting multiple systems [OR 2.70, 95% confidence interval (CI) 1.30–5.63, *p* = 0.018], the skin (OR 2.23, 95% CI 1.33–3.75, *p* = 0.007), and the vertebral, anorectal, cardiovascular, tracheo-esophageal, renal, and limb association (VACTERL) (OR 1.51, 95% CI 1.16–1.96, *p* = 0.007). These data provided genetic evidence supporting current manufacturer advice to avoid the use of PCSK9 inhibitors during pregnancy [92,93].

Noseda et al. used the World Health Organization global pharmacovigilance database, to search for signals of disproportionate reporting for pregnancy outcomes with alirocumab and evolocumab [94]. They found 45 reports of exposure to evolocumab (N = 31) and alirocumab (N = 14) in pregnancy, most of them originated in Europe (N = 25, 55.6%). According to the authors, drug exposure occurred during pregnancy in 36 (80.0%) safety reports, via paternal exposure during pregnancy in four (8.9%), during lactation in three (6.7%), and in two safety reports the time of drug exposure remained unknown. Twenty safety reports (57.8%) merely reported drug exposure, while 19 (42.2%) also reported pregnancy outcomes, but without specific maternal toxicities or patterns of birth defects. Thus, the authors concluded that there are no signals of increased reporting of spontaneous abortion [94].

**Lipoprotein apheresis:** Lipoprotein apheresis (LA) involves the physical removal of lipoproteins primarily LDL and lipoprotein (a) from the bloodstream. Different methods of extracorporeal LDL-apheresis exist, such as immunoadsorption, dextran sulfate adsorption, and double plasma filtration, which are all-effective and depend on institutional preference.

Apheresis is a well-established extracorporeal intervention to treat patients suffering from familial hypercholesterolemia and coronary heart disease if all other lipid lowering approaches such as fat modified diet, HMG-CoA reductase inhibitors in combination with sterol transport blockers (e.g., ezetimibe) or ion-exchange resins provide insufficient reduction in LDL cholesterol [95,96]. Of note, studies have shown that apheresis improves blood rheology, reduces oxidative stress parameters, improves endothelial function and downregulates adhesion molecules and inflammatory cytokines [97,98,99].

Although LA is reported as the most effective therapy to control lipid levels in women with familial hypercholesterolemia or atherosclerotic cardiovascular disease (ASCVD) during pregnancy, only a limited number of case reports has been published. Pregnant women with familial hypercholesterolemia should be offered weekly or fortnightly LA during pregnancy [100]. To minimize LDL-C exposure, LA should be offered during pregnancy, and statins plus other lipid-lowering therapy may possibly be restarted from the second trimester.

Ogura et al. reported ten successful deliveries in seven women with homozygous familial hypercholesterolemia and compared the clinical outcomes between patients who received LA during pregnancy and those who did not [101]. After exclusion of pregnant women who died during pregnancy either due to refusal of LA intervention or poor adherence to LA, early initiation of LA from childhood and good adherence to it during pregnancy resulted in the delivery of healthy infants without adverse effects.

Bláha et al. [102] presented case reports of six women with homozygous familial hypercholesterolemia and their 13 pregnancies (nine successful, three abortions, and one interruption). Of the nine successful pregnancies, two cases were treated by LDL-apheresis indicated that LDL-apheresis plays an important role in the management of pregnancy in homozygous familial hypercholesterolemia [102].

Furthermore, there is some evidence that LA may reduce the risk of cardiovascular events in pregnant women with severely elevated Lp(a) levels, cardiovascular disease, and a high risk of preeclampsia. Recently, Marlȩga-Linert J et al. presented a case of a pregnant woman with severely elevated Lp(a), two previous episodes of the acute coronary syndrome and multivessel coronary disease treated with long-term LA before pregnancy, and a high risk of preeclampsia [103]. A bi-weekly LA therapy was re-initiated at 21 weeks’ gestation by which the 70% of the serum Lp(a) concentration was removed and low-density lipoprotein-cholesterol (LDL-C) levels were reduced by 60%. The pregnancy lasted until 36th week, when severe preeclampsia prompted an emergency cesarean delivery. According to the authors, this case indicated the benefits of LA in preventing atherosclerotic CVD progression during pregnancy, its potential influence on uteroplacental circulation, and prolongation of pregnancy for the best possible intrauterine fetus development [103].

**Table 2:** Characteristics and outcomes of the clinical studies.

Study	Study Design	Exposure	Exposure Time	Outcomes
**Fibric acid derivatives**				
Sunman et al., 2012 [55]	Case report	Fenofibrate	From the beginning of fertilization	No harmful effects on fetal development.
Whitten et al., 2011 [56]	Case report	Fenofibrate	During pregnancy	No harmful effects.
**Omega-3 fatty acids**				
Nguyen et al., 2021 [58]	Case report	Lovaza	Not available.	No harmful effects.
**Statins**				
Manson et al., 1996 [64]	Cases	Lovastatin, Simvastatin	First trimester in 89% of cases	85% normal outcomes (births), 4.0% congenital anomalies, 8.0% spontaneous abortions, 1.0% fetal deaths/stillbirths, 2.0% miscellaneous adverse outcomes.
Edison et al., 2004 [65]	Cases	Lovastatin, Simvastatin, Atorvastatin, Cerivastatin	First trimester	Nine cases of malformation in infants (limb deficiencies and central nervous system defects).Τhe adverse outcomes at birth were associated with a lipophilic statin.
Pollack et al., 2005 [66]	Cases	Simvastatin and/or Lovastatin	During pregnancy	Six congenital anomalies (chromosomal translocation, trisomy 18, hypospadias, duodenal atresia, cleft lip, and skin tag).
Petersen et al., 2008 [67]	Cases	Atorvastatin, Simvastatin, Pravastatin, Cerivastatin	First trimester	Congenital anomalies (7 congenital heart defects, 2 cleft lip or palate defects, 2 neural tube defects, 2 other miscellaneous defects) and 9 birth defects (5 congenital heart defects, 2 cleft lift lip defects, 2 other miscellaneous defects).
Taguchi N et al., 2008 [68]	Cohort study	Atorvastatin, Simvastatin, Pravastatin, Rosuvastatin	First trimester	There was no difference in the rate of major malformations between the two groups 2.2% vs. 1.9% (1/46 live birth in statin group vs. 1/52 live birth in control group, *p* = 0.93). No statistical significant differences between the statin and control group concerning live births (71.9% vs. 81.2%), spontaneous abortions (14: 21.9% vs. 11: 17.2%), therapeutic abortions (3: 4.7% vs. 0: 0%) and stillbirths (1: 1.5% vs. 1: 1.6%).
Winterfeld et al., 2013 [69]	Cohort study	Simvastatin, Atorvastatin, Pravastatin, Rosuvastatin, Fluvastatin, Cerivastatin	First trimester	No teratogenic effect of statins. No statistically significant difference in pregnancy outcomes (rate of major birth defects, median gestational age at birth and birth weight) between statin-exposed and matched non-exposed pregnancies. The premature birth was more frequent in exposed to statins pregnancies (16.1% versus 8.5%; OR 2.1, 95% CI 1.1–3.8, *p* = 0.019).
Bateman B.T. et al., 2015 [70]	Cohort study	Atorvastatin, Simvastatin, Lovastatin, Pravastatin, Fluvastatin, Rosuvastatin	First trimester	In the unadjusted analysis, the prevalence of malformations in the offsprings was 6.34% compared with 3.55% in those of non-statin exposed pregnant women in the first trimester (relative risk (RR) 1.79, 95% confidence interval (CI) 1.43 to 2.23). After adjusting for confounders, this risk was decreased (1.07, 0.85 to 1.37).
Ofori et al., 2007 [71]	Registry-based study	Atorvastatin, Fluvastatin, Lovastatin, Pravastatin, Simvastatin,	First trimester	Among women with a live birth, the rate of congenital anomalies was 3/64 in group A (4.69%; 95% CI 1.00, 13.69), 3/14 in group B (21.43%; 95% CI 4.41, 62.57) and 7/67 in group C (10.45%; 95% CI 4.19, 21.53). The adjusted OR for congenital anomalies in group A compared with group C was 0.36 (95% CI 0.06, 2.18). *group A*: women prescribed statins in the first trimester. *group B*: women prescribed fibrate/nicotinic acid in the first trimester. *group C*: women used statins between one year and one month before conception, but not during pregnancy.
Colvin et al., 2010 [72]	Registry-based study	Atorvastatin, Simvastatin	First trimester	Among the records of births, the researchers identified 51 records of live births whose mothers have been exposed to atorvastatin (n = 33) and simvastatin (n = 18) during the first trimester. No congenital anomalies were reported in the study.
Toleikyte I et al., 2011 [73]	Registry-based study	Not available	Not available	Pregnant women with familial hypercholesterolemia exposed to lipid-lowering drugs do not appear to have a higher risk of preterm delivery or of having infants with low birth weight or congenital malformations than women in general.
**Proprotein convertase subtilisin/kexin type 9 (PCSK9) inhibitors**				
Vuignier Y et al., 2021 [90]	Case report	Rosuvastatin, Ezetimibe, Alirocumab	During pregnancy	Both the mother and infant were discharged without any notable complications.
Suzuki et al., 2024 [91]	Case report	Evolocumab	Evolocumab was administered at 31 and 35 weeks of gestation	Both the mother and infant were discharged without any notable complications.

## 5. Conclusions

In summary, pregnancy is a unique physiological state in which distinct alterations in lipid metabolism are occurring to meet the energy needs for fetal growth and development. Without doubt, dyslipidemia in pregnancy is associated with adverse pregnancy outcomes that enhance the risk of clinical ASCVD events in later life. However, the available guidelines on the management of lipid disorders during pregnancy and the postpartum period concern mainly lifestyle and dietary interventions and to a lesser extent pharmacological agents. The management of dyslipidemia during pregnancy requires personalized and multidisciplinary care (e.g., cardiologists, obstetricians, dietitians), which needs to be utilized to optimize long-term cardiometabolic health of pregnant women. Further research and clinical trials are needed in this special patient population to establish evidence-based protocols and clinical guidelines to guide effective therapeutic options.

## Figures and Tables

**Table 1:** Alterations in lipid metabolism during pregnancy.

	Months of Pregnancy		
	1st–2nd	3rd–7th	8th–9th
Fat accumulation	↑	↑	↓
Total Cholesterol	↓	↑	↑
Triglycerides	↓	↑↑	↑↑
LDL-Cholesterol	↓	↑	↑
HDL-Cholesterol	-	↑	-

## Data Availability

Not applicable.
